# An Outdated Notion of Antibody Specificity is One of the Major Detrimental Assumptions of the Structure-Based Reverse Vaccinology Paradigm, Which Prevented It from Helping to Develop an Effective HIV-1 Vaccine

**DOI:** 10.3389/fimmu.2014.00593

**Published:** 2014-11-18

**Authors:** Marc H. V. Van Regenmortel

**Affiliations:** ^1^CNRS, Biotechnologie des Interactions Moleculaires, IREBS, School of Biotechnology, ESBS, University of Strasbourg, Illkirch, France

**Keywords:** structure-based reverse vaccinology, HIV-1 vaccines, antibody polyspecificity, paradigm, immunological explanations

## Abstract

The importance of paradigms for guiding scientific research is explained with reference to the seminal work of Karl Popper and Thomas Kuhn. A prevalent paradigm, followed for more than a decade in HIV-1 vaccine research, which gave rise to the strategy known as structure-based reverse vaccinology is described in detail. Several reasons why this paradigm did not allow the development of an effective HIV-1 vaccine are analyzed. A major reason is the belief shared by many vaccinologists that antibodies possess a narrow specificity for a single epitope and are not polyspecific for a diverse group of potential epitopes. When this belief is abandoned, it becomes obvious that the one particular epitope structure observed during the crystallographic analysis of a neutralizing antibody–antigen complex does not necessarily reveal, which immunogenic structure should be used to elicit the same type of neutralizing antibody. In the physical sciences, scientific explanations are usually presented as logical deductions derived from a relevant law of nature together with certain initial conditions. In immunology, causal explanations in terms of a single cause acting according to a law of nature are not possible because numerous factors always play a role in bringing about an effect. The implications of this state of affairs for the rational design of HIV vaccines are outlined. An alternative approach to obtain useful scientific understanding consists in intervening empirically in the immune system and it is suggested that manipulating the system experimentally is needed to learn to control it and achieve protective immunity by vaccination.

“It makes no sense to do the same thing over and over again and expect a different result.”

Albert Einstein

## Introduction

The development of science is promoted when scientists adhere to so-called paradigms that correspond to theoretical presuppositions and assumptions that guide the lines of investigation they pursue. When trying to solve a particular scientific puzzle, for instance how to develop an effective HIV-1 vaccine, the paradigm will also commit scientists to using particular experimental approaches and tools believed to be essential for finding a solution to the problem.

The importance of paradigms in guiding scientific research was proposed by Thomas Kuhn (1) in his influential book “*The Structure of Scientific Revolutions*” published in 1962. Unfortunately, he never clearly defined the concept of paradigm and he used the term in various ways, for instance, to refer to a collection of procedures and ideas that instruct scientists what to believe and how to work (2). Kuhn later conceded that the term paradigm had become hopelessly overused because it came to signify virtually any dominant idea that binds a scientific community together. He subsequently recommended that *paradigm* be replaced by *exemplar* to mean exemplary instances of successful puzzle-solutions for scientific problems. An exemplar captures the way in which a theory or model is believed to solve a problem while at the same time defining, which new problems could be addressed in a similar way. However, the term paradigm was never abandoned.

Kuhn argued that when scientists in the course of their work obtain results that contradict the theory or hypothesis that gave rise to a paradigm, they do not conclude that the paradigm has been refuted and must be abandoned. Scientists, therefore, do not follow the injunction of Karl Popper that their aim should be to try to disprove or falsify their theories rather than prove them. Popper maintained that observations are never able to prove a theory but can only sometimes logically refute a mistaken theory (3). He argued that when scientists obtain reproducible results that are at odds with their working hypothesis, they are logically obliged to accept that the hypothesis has been falsified and they should therefore abandon it (4). Kuhn disagreed and claimed that this is not the way scientists behave because their main commitment is not to test or seek to confirm the implicit theories and hypotheses that underlie the paradigms they adhere to. Scientists in fact tend to ignore anomalous results and will devise new *ad hoc* hypotheses in an effort to explain away apparent contradictions between theory and experimental observations. Kuhn further claimed that science can make progress only if scientific communities remain committed to their shared theoretical beliefs and experimental techniques and do not abandon a paradigm or hypothesis as soon as incompatible results are obtained (5). Only if troublesome anomalies keep accumulating over many years may scientists eventually start questioning their presuppositions and lose their confidence in a given paradigm. This could then usher a scientific revolution that occurs when a paradigm is superseded by a new one and gives rise to a paradigm shift. Periods of so-called normal science are then replaced by a short period of revolutionary science (1).

In HIV vaccine research, there is evidence that several prevalent paradigms have not helped the development of an effective vaccine (6–8). One such paradigm, which gave rise to the strategy known as structure-based reverse vaccinology (RV) (9) was pursued vigorously for more than a decade although it did not lead to the development of an effective HIV-1 vaccine. The theoretical underpinnings of this paradigm have been discussed previously because they illustrate the need for investigators to question the implicit underlying assumptions that make them pursue unfruitful lines of investigation (10, 11). Only when the presuppositions or hypotheses that gave rise to unsuccessful paradigms are shown to be invalid will investigators become aware that a paradigm shift is required in a particular scientific field (8).

## Structure-Based RV Paradigm in HIV-1 Vaccine Research

The approach known as RV was introduced in the field of bacterial vaccines by Rino Rappuoli (12, 13) and refers to the strategy of predicting potential vaccine immunogens using bioinformatics analyses of entire bacterial genomes in order to identify all the surface-exposed proteins that a bacterial pathogen is able to express. The strategy is called RV because investigators operate in a reverse manner, i.e., starting from the genome rather than from the organism, to discover, which bacterial proteins should be studied as potential vaccine immunogens. This allows hundreds of bacterial proteins to be identified as candidate immunogens even when bacteria cannot be cultivated and bacterial extracts cannot therefore be fractionated to establish empirically which proteins are able to induce a protective immune response.

In virology, RV has a different meaning and refers to a strategy, which attempts to generate a vaccine from a knowledge of protective antibodies (Abs) rather than from the usual reverse task of generating such Abs by immunization with a vaccine (9, 14). It was suggested that effective vaccine immunogens might be discovered by exploring the interaction of anti-HIV-1 neutralizing (n)Abs with HIV-1 envelope (Env) spikes, using X-ray crystallography of Env–Ab complexes. This expectation was based on the assumption that once the 3D structure of a broadly neutralizing monoclonal antibody (bnMab) bound to an Env epitope had been elucidated, it would be possible to use the bnMab as a template to reconstruct its complementary epitope outside the context of the natural Env antigen, using structure-based design. It was further hypothesized that this reconstructed epitope designed to fit the bnMab would possess the immunogenic capacity of inducing a polyclonal Ab response with the same neutralizing capacity as the bnAb used as template. The assumption was that if an HIV-1 epitope is able to bind an Ab, it will also possess the immunogenic capacity to elicit the same type of Ab in an immunized host. That this is not necessarily the case, however, is demonstrated by the common observation that when a peptide fragment of a protein is able to bind Abs raised against the protein, the peptide will frequently be unable to elicit Abs that react with the native protein (15).

Since the RV approach used to develop bacterial vaccines has been highly successful (13) whereas, the RV approach used in the HIV-1 vaccine field has failed so far (10, 16, 17) it has been suggested that the two approaches should be clearly differentiated and could be called genome-based RV and structure-based RV, respectively (18).

## Unwarranted Assumptions of the Structure-Based RV Paradigm

The structure-based RV approach suffers from several unwarranted expectations that jeopardized the ability of developing an effective HIV-1 vaccine. It did not clearly distinguishing between antigenicity and immunogenicity (12, 19) and analyzed epitopes in antigens and paratopes in Abs solely in terms of which amino acids in the two partners made contact with each other (20). As a result, little attention was given to the fact that the binding activity of an Ab often depends on structural features distant from the paratope itself (21, 22) and that residues in the antigen that are not in contact with the paratope may nevertheless be able to affect the binding activity and the immunogenic activity of epitopes (23, 24). Such findings confirm an insight reached years ago that paratopes and epitopes defined in terms of contact residues often differ from the binding sites defined in functional assays (25). The structures visualized in Ab-antigen complexes also tend to differ from the structures of the binding sites in the free molecules, before they have been altered by the mutual adaptation and induced fit that occur when the two partners interact (26, 27). This means that the particular antigenic structure revealed in an Ab–antigen complex does not necessarily correspond to the immunogenic structure that was recognized by B-cell receptors (BCRs) during the immunization process and which therefore is often presumed to be needed in the vaccine immunogen.

In an earlier review (11), more than 50 original science publications were mentioned, which attempted to reconstruct active HIV-1 epitopes using conformational constraints, protein scaffolds, and other structure-based engineering approaches. Although some of the engineered epitopes possessed increased antigenicity and reacted better with bnMabs, none of them were found to be effective vaccine immunogens, illustrating the shortcomings of the structure-based RV approach (28–32).

## Overlooking the Need for Antibody Affinity Maturation to Obtain Effective Anti HIV-1 Neutralizing Abs

Another unjustified hypothesis of the structure-based RV approach was that the HIV-1 epitopes recognized by the matured bnMabs that are present in HIV-1 infected individuals after a lengthy process of Ab affinity maturation will be able to trigger a protective immune response in naïve individuals. However, studies involving the deep sequencing of all the HIV-1 Abs present in the serum of infected individuals demonstrated that the initial immunogen triggering the Ab affinity maturation process that leads to neutralizing Abs usually recognizes a germline version of BCRs that differs considerably from the BCRs corresponding to mature bnAbs (33, 34). This means that the epitopes engineered by structure-based RV to mimic HIV-1 epitopes recognized by mature bnMabs are unlikely to be effective vaccine immunogens because they are mostly unable to recognize the germline BCRs present in naïve individuals (35–38).

The extent of affinity maturation observed in HIV-1 bnAbs is much more extensive than the 5–10% mutation frequency in the Ab hypervariable regions observed with Abs directed to other viruses (37–39) and this characteristic feature of HIV-1 bnAbs together with the enormous antigenic variability of the virus explains why the structure-based RV approach in the case of HIV-1 presented unsurmountable challenges compared to other viral vaccines (36, 40, 41). HIV-1 elicits bnAbs only in a minority of infected individuals after several years of infection and attempts are currently made to unravel the mutational pathway that leads from germline BCR ancestors to mature Abs (42). The goal is to identify various immunogens capable of stimulating successive B-cell responses through multiple rounds of antibody maturation processes (43, 44). This is clearly no mean task since large numbers of maturation pathways are likely to exist (45–47).

Current attempts to modify Env epitopes so that they are able to bind germline BCRs or maturation intermediates depart from the original structure-based RV paradigm because they no longer attempt to directly transform epitopes of known structure recognized by mature bnMabs into immunogens capable of eliciting similar bnAbs (46–48). This new approach represents a new paradigm based on the germline/maturation hypothesis (34), which assumes that it may be possible to discover effective HIV-1 vaccine immunogens that bind putative germline antibody predecessors of known HIV-1 bnAbs although they do not bind the highly somatically mutated bnAbs themselves (49).

In recent years, increasing numbers of bnMabs have been isolated from HIV-1 infected individuals (50–52) and it has been suggested that these Mabs provide valuable molecular information that could inform HIV-1 vaccine design (53, 54). Such bnMabs may be of value for passive immunotherapy since they could provide sterilizing immunity to humans and non-human primates when they are administered prior to viral challenge (55, 56). However, their usefulness does not at present extend to the vaccine field since we have no idea how Abs that possess the protective capacity of such bnMabs can be induced by vaccination (11). There is also considerable evidence that a protective immune response requires the combined neutralizing activities of several Abs that target different non-overlapping HIV-1 Env epitopes (57) as well as Abs that act in synergy (58–60). What may be required, therefore, is to find immunogens able to elicit classical types of protective, polyclonal immune responses rather than elusive immunogens that would elicit single Ab specificities endowed with the exceptional neutralizing capacity of individual bnMabs such as the well-studied b12, VRC01, PG9, or PG16 bnMabs (61–63).

## Reliance on Reductionist Thinking: Another Pitfall of the Structure-Based RV Paradigm

Another pitfall of the structure-based RV paradigm is its reliance on reductionist thinking (11). Reductionism has been prevalent in molecular biology for half a century and is still popular today because it has been very successful for dissecting biological systems into their constituent parts (64, 65). The reductionist mindset made immunologists accept that the biological activities of Abs could be explained by their 3D structures and that the immunogenic potential of a viral epitope could be deduced from its antigenic properties. Biological immunogenicity was thereby reduced to chemical antigenicity, which is a variation of the claim that biology can be reduced to chemistry (66). Such a claim fails to recognize that the protection achieved by vaccination is a biological phenomenon that has meaning only in the context of an entire organism since organs, tissues, or molecules cannot be vaccinated. Protection always results from a complex network of dynamic interactions between pathogen, host, and immune system and it cannot be satisfactorily understood when innumerable, individual molecular interactions are analyzed separately.

The use of Mabs also introduces a reductionist bias in the analysis of protein antigenicity and immunogenicity because it leads investigators to focus on artificial boundaries between overlapping epitopes and to ignore the fact that the apparent immunological specificity of a Mab very much depends on the selection process that was used to obtain it. Since a Mab is always polyspecific, the fact that it binds, for instance, to one particular peptide of the membrane proximate external region (MPER) of HIV-1 gp41 simply reflects the fact that the Mab was selected for its ability to bind to that peptide. Such a Mab may, however, recognize better a more complex epitope of gp41 that could actually have been the immunogen that gave rise to the Mab. If it is assumed that the Mab was elicited by the linear MPER peptide epitope because it binds to it, this may induce investigators to only investigate peptides as possible vaccine immunogens, a choice, which is likely to be self-defeating (15, 67).

## Structure-Based RV Paradigm Suffers from Another Major Misguided Assumption, Namely an Outdated Notion of Ab Specificity

According to this view, which is usually not explicitly acknowledged, a Mab that would bind for instance to an epitope on the HIV-1 Env surface is believed to be able to recognize only a single defined target area (the so-called complementary epitope of the Mab) corresponding to the surface residues found to be in contact with paratope residues in the Ab–antigen complex. Although the belief that Mabs are monospecific for a single epitope is no longer held and was shown to be invalid already 30 years ago (68–74) many vaccinologists have been slow to accept that there is no single intrinsic or specific epitope for any Ab but only a diverse group of potential epitopes able to bind to it with various degrees of fit (10, 75, 76). It seems that the failure to recognize that Abs are always polyspecific and possess considerable plasticity, promiscuity, degeneracy, and cross-reactive potential has made the structure-based RV approach appear more plausible to many investigators (10).

It is now well-established that most Abs derived from Ab germline genes and expressed before any antigenic stimulation are highly polyreactive and react with a wide variety of autoantigens such as DNA, cytoskeleton proteins, nuclear antigens, and carbohydrates as well as numerous bacterial and viral antigens (70, 77–81). The various mechanisms that allow a polyspecific Ab to recognize a multiplicity of epitopes and antigens have been elucidated (82). Once investigators realize that the epitope structure observed in a bnMab–HIV-1 Env complex is only one of the many epitopes that could be accommodated by what is always a polyspecific Ab, they have no difficulty to accept that any one of these epitopes could correspond to the immunogen that gave rise to the Mab. They will then no longer assume that the one epitope structure observed in the crystallographic analysis of an antigen complex must necessarily reveal, which immunogenic structure should be used to elicit such a bnAb (10).

Furthermore, the fact that Abs are not only polyspecific but are also heterospecific, i.e., able to react more strongly with other antigens than with the immunogen used for eliciting the Ab, also helps to clarify why antigenic and immunogenic properties are not always simultaneously present in the same region of a protein (76). The immunogenic capacity of an epitope to induce heterospecific Abs that do not react with the protein used for immunization demonstrates that immunogenicity need not be accompanied by an antigenic reactivity that enables the epitope to bind to the induced Abs. Similarly, the antigenic reactivity of a viral protein revealed by its ability to bind a given polyspecific Ab is also not necessarily accompanied by an immunogenic capacity to induce that same Ab in a particular immune system (76).

Although the advent of Mabs has completely transformed our ability to dissect immune responses to proteins, their utilization has also introduced a bias in the antigenic analysis of viruses because investigators tend to focus on artificial boundaries between overlapping epitopes and overlook the fact that the surface of a protein is an antigenic and functional continuum (83). When an nMab has been isolated, it tends to become associated with a unique, discrete epitope, which leads to the expectation that a vaccine containing this epitope will induce similar nAbs. However, most protective immune responses are polyclonal and involve the collective and synergistic neutralizing activities of Abs directed to different epitopes (61). When the therapeutic efficacy of mixtures of HIV-1 neutralizing Mabs was tested in HIV-1 infected humanized mice, it was found that mixtures of five Mabs were more effective than single Mabs or mixtures of three Mabs (84). Mixtures of five Abs suppressed viral loads below the level of detection and also failed to select escape viral mutants. However, chronically HIV-1 infected humans from whom bnAbs were isolated do not appear to benefit from such Abs for controlling virus replication (17, 85, 86), nor do HIV-1 infected long-term progressors compared to non-progressors (87). The role of Abs in controlling chronic HIV-1 infection therefore remains an issue that should be further investigated.

Many unsuccessful attempts have been made to elicit by immunization bnAbs similar to potent bnMabs isolated from HIV-1 infected individuals (17, 88). The past research emphasis on unraveling the neutralization mechanisms of individual bnMabs did not help because it did not provide any information on which immunogens were capable of eliciting the different types of bnAbs. The poor success rate so far in discovering even one such effective HIV-1 vaccine immunogen by structure-based RV is certainly a cause of concern. As discussed elsewhere (8), there is a clear need to study new vaccine immunogens and new methods to induce strong mucosal antibody responses using for instance specific adjuvants. Other markers than viral load and CD4 T cell counts for assessing vaccine efficacy should also be investigated (8).

It is nowadays commonly claimed that rational design offers the best prospects for developing an HIV-1 vaccine and that this approach is superior to the empirical screening and trial-and-error strategies used in the past. When it is claimed that “rational design represents the only approach that can elevate vaccine research from an empirical exercise to a scientific discipline” (89), the essential contribution of empirical trials to vaccine development is actually denigrated as if trial-and-error experimentation were not an entirely rational enterprise (10, 11, 19). When authors discuss the rational design of an HIV-1 vaccine (90, 91) they only refer to studies that improve the degree of complementarity in a single epitope–Mab pair and they do not clarify how an improved antigen could actually be “designed” to also become an immunogen capable of inducing protective Abs. Optimizing the binding activity of a viral antigen by structure-based design using a single Mab as a template is certainly feasible but this is not equivalent to immunogen design, which requires the intentional optimization of numerous factors extrinsic to the epitope–paratope recognition such as the various cellular and regulatory mechanisms of the host that exist only in the context of the vaccinated host and control the generation of neutralizing Abs (11, 92). Antigen design is simply masquerading as immunogen design when it is assumed that an improved viral antigen will also be an effective vaccine immunogen capable of inducing a protective immune response. The so-called rational design of vaccine immunogens by mimicking the rational approach used in drug design (10, 93, 94) is not feasible without extensive empirical clinical trials of vaccine candidates and to suggest otherwise contradicts the well-established empirical nature of vaccine science. The concern that too much funding may be diverted to empirical clinical trials at the expense of basic structure-based HIV vaccine research (95) seems unwarranted since the lion share of current funding is devoted to structural studies whereas, the required small scale clinical trials based on innovative paradigm concepts remain poorly funded (8).

## Causes, Explanations, and Understanding in Vaccinology

The selection of a paradigm to guide the empirical search for an HIV-1 vaccine always depends on framing hypotheses about causal mechanisms, which could provide a plausible explanation for a possible successful vaccine. Most scientific explanations take the form of a causal mechanistic explanation, which means that causes, explanations and understanding are usually intimately linked (96, 97). It is relevant therefore to analyze how these three terms are used in vaccinology.

In the physical sciences, explanations are usually presented as logical deductions derived from one or a few relevant laws of nature, together with certain initial conditions. However, this is not feasible in the biological sciences because of the absence of universal biological laws (11, 98). Since the probability of a biological event is always affected by a very large number of causal factors, causal links become diluted and it is usually not possible to provide an explanation in vaccinology in terms of a single cause.

The reductionist dissection of the immune system into its components severs the dynamic connections that link the parts of a biological complex system in a functionally integrated manner. This allows any level in the resulting biological complexity to be the starting point of a causal analysis, provided a certain state of affairs is considered to be in need of an explanation. As a result, explanations are usually framed in terms of a complex type of probabilistic causality that attempts to take into account the numerous factors that together contribute to an effect in a given biological context. This means that the classical notion of causality is of limited value for providing explanations in immunology (11, 96). The contribution of one causal factor in a complex multicausal biological system can actually only be investigated by altering that factor experimentally and assessing whether the observed effect is then no longer the same in a given context (94). This has led to the suggestion that many biological phenomena may be too complex to be comprehended or explained by human intelligence (99).

Attempts to achieve understanding in immunology often start with information about observed effects and by an awareness that certain phenomena share underlying similarities, which then leads one to propose a theory or hypothetical model to explain them. The structure-based RV paradigm is one such model, which assumes that bnMabs that are isolated from HIV-1 infected individuals and recognize particular Env epitopes are likely to have been elicited by these epitopes. When the model was tested by assessing the immunogenicity of the epitopes recognized by different bnMabs, it was found that the results did not fit the model and that no bnAbs were elicited (29, 30, 100). This should remind us that a proposed explanatory model does not predict the data to which it is fitted since the model is actually chosen to fit the data and it makes no sense to fit what one wants to explain (101). Understanding consists of knowledge about relations of dependence and should make it possible to derive inferences about the consequences of our interventions and give us an ability to predict and control phenomena. Unfortunately, it seems that scientists tend to overestimate the detail and depth of their understanding, which often consists of nothing more than an informed guess about the future prospects of their scientific work. This leads to a frame of mind that has been called an “illusion of depth of understanding in science” (102). In the absence of experimental data that back their explanatory model or paradigm, scientists do not choose which theory to accept but choose which theory they are actually going to work with. Scientists are then driven by the promise of future understanding rather than by past convincing explanatory evidence and such an expectation is easily influenced by wishful thinking about their pet theories (102).

Since understanding a phenomenon is ultimately displayed by: (1) making right predictions, (2) successfully intervening in a system, and (3) answering explanation-seeking questions about it, the inability to do any of these things is a clear indication that alternative explanatory hypotheses and paradigms are required. As far as HIV-1 vaccines are concerned, our ignorance of why all Env immunogens investigated so far have been unable to induce adequate levels of potent protective Abs is a clear indication that we do not understand the complex mechanisms that are involved in achieving protective immunity against HIV-1.

Although the complexity of the immune system may prevent us from identifying all its internal regulatory mechanisms, it is by comparing the various ways of manipulating the system using empirical experimentation that we may eventually control it and achieve protective immunity by vaccination (11).

Empirical, scientific knowledge is based on experimental and observational facts and on the rule that “nothing trumps experience.” However, it has been argued that empirical evidence only allows scientists to draw plausible but tentative conclusions when the obtained results make it possible to successfully control and manipulate the experimental system under investigation (103, 104). If this does not happen, empiricism dictates that we should investigate additional constituents of the complex biological systems we study until we improve our ability to predict the results of our experimental interventions. It is indeed the ability of investigators to successfully intervene in a material system that gives them the knowledge needed to manipulate and control it.

Since the aim of biologists is both to explain and control biological phenomena, explaining goes hand-in-hand with intervening and it has been suggested that “explanations in biology are always obtained through direct intervention on models of the phenomenon to be explained” (105). It is important to recognize that such a view is at odds with the widespread expectation that we will succeed in developing an effective HIV-1 vaccine only when we have significantly increased our general knowledge of basic immunology and of HIV-1 antigenic structure (11). This means that we need to interfere with the material world in order to obtain empirical knowledge about it and that our scientific understanding increases when we are able to successfully manipulate the system we investigate (106).

## Conclusion

There is evidence that the popular structure-based RV paradigm used in HIV-1 vaccine research has not been helpful for developing an effective HIV-1 vaccine. Some of the reasons for this lack of success are summarized, one of them being the failure to recognize that all Abs as well as bnMabs isolated from HIV-1 infected individuals are always polyspecific and able to bind to a variety of related and unrelated epitopes. Since the epitope structure identified by X-ray crystallography of a bnMab–HIV-1 Env complex is only one of several epitopes that could be accommodated by the Mab, there is no reason to assume that this epitope of known structure must correspond to the immunogen that elicited the antibody.

Recently, one of the major groups committed to the structure-based RV paradigm reported a study of bnMabs that recognize a high-mannose epitope patch centered on the N 332 residue on HIV Env. They demonstrated that these Abs actually did not have a single defined target point at N 332 but were in fact polyspecific and able to bind various glycan patches as well as a glycan site located at N334 (107). For the first time, these authors admitted that antibody polyspecificity was a relevant concept in HIV-1 vaccine research and they claimed that their results represent an extension of the concept of antibody promiscuity and degeneracy that has been widely accepted in immunology for many years (10, 68, 73–75). They also concluded that polyclonal Abs are more effective for neutralizing viruses than individual Mabs and that the polyspecificity of vaccine-induced Abs should receive increased attention. The new insights arrived at by these authors might in time alter the expectations of the proponents of the structure-based RV paradigm and diminish their reliance on structural data derived from the study of individual bnMabs.

The structure-based RV paradigm has been followed by several large networks of investigators who operate under the strong leadership of principal investigators (108). This situation leads to considerable built-in inertia and does not encourage funding agencies to back large numbers of high-risk projects based on alternative innovative paradigms that could diversify the vaccine strategies that are investigated (8, 108). These issues are currently receiving increased attention and it is hoped that this will in due course lead to increased funding for new original science based on novel paradigms and that it will stimulate a rigorous evaluation of existing HIV vaccine programs (108).

## Conflict of Interest Statement

The author declares that the research was conducted in the absence of any commercial or financial relationships that could be construed as a potential conflict of interest.
